# Injecting-related trust, cooperation, intimacy, and power as key factors influencing risk perception among drug injecting partnerships

**DOI:** 10.1371/journal.pone.0217811

**Published:** 2019-05-31

**Authors:** Meghan D. Morris, Erin Andrew, Judy Y. Tan, Lisa Maher, Colleen Hoff, Lynae Darbes, Kimberly Page

**Affiliations:** 1 Department of Epidemiology & Biostatistics, University of California, San Francisco, CA, United States of America; 2 Nursing Science, University of Pennsylvania, Philadelphia, PA, United States of America; 3 Center for AIDS Prevention Science, University of California, San Francisco, San Francisco, CA, United States of America; 4 Kirby Institute, UNSW Sydney, Sydney, Australia; 5 Center for Research and Education on Gender and Sexuality, San Francisco State University, San Francisco, CA, United States of America; 6 Center for Sexuality and Health Disparities, Department of Health Behavior & Biological Sciences, School of Nursing, University of Michigan, Ann Arbor, MI, United States of America; 7 Department of Internal Medicine, University of New Mexico, Albuquerque, NM, United States of America; University of Cyprus, CYPRUS

## Abstract

Sharing of injection drug use paraphernalia is a dyadic process linked to the transmission of HIV and hepatitis C virus (HCV). Despite this, limited research exists identifying specific dyadic interpersonal factors driving injecting partners’ engagement in needle/syringe and ancillary injecting equipment sharing among young adults. Using semi-structured in-depth interview data collected between 2014 and 2015 from twenty-seven people who inject drugs (PWID), we applied an inductive approach to identify key injection drug-related interpersonal factors and developed a conceptual model integrating the findings based on interdependence theory. Interactions between injecting partners resulted in varying levels of injecting-related trust, cooperation, intimacy, and power. These factors interacted to collectively influence the type and level of risk perceived and enacted by injecting partners. The relationship between these injecting-related interpersonal factors, on the one hand, and risk perception on the other was dynamic and fluctuated between actions that protect the self (person-centered) and those that protect the partnership (partnership-centered). These findings indicate that the interpersonal context exerts substantial influence that shapes risk perception in all types of injecting partnerships. Partnership-focused prevention strategies should consider the dynamics of trust, cooperation, intimacy, and power, in characterizing dyadic risk perceptions and in understanding risky injecting practices among PWID.

## Introduction

In the United States, hepatitis C virus (HCV) infection continues at epidemic levels with people who inject drugs (PWID) experiencing the greatest burden of disease [1]. In many areas upward of 60% of PWID are infected with HCV and incidence remains high at 5–40% annually, with young adult PWID (<30) as the group with highest incidence [2–4]. Among people who inject drugs together, risk is typically characterized by identifying the factors associated with behaviors that increase exposure to HCV or HIV, such as sharing of injecting equipment. Sharing of needles/syringes and ancillary injecting equipment (e.g., cookers, cottons, and mixing containers), a dyadic process occurring between at least two people, are the most efficient modes of HCV transmission and are common behaviors, with 40–70% of PWID in the U.S. reporting recent sharing [5, 6].

The reasons for sharing needles and injecting equipment are multiplex. Although individual factors (such as knowledge, perceived risk, and perceived sense of control) undoubtedly influence injecting and sharing behaviors [7, 8], a growing body of evidence has drawn attention to the important role of structural, social, and economic factors. For example, the well-established “risk environment” framework applied by Rhodes et al. has produced key insights about the role of policing, economic instability, social disruption, and prevention service coverage on injecting behaviors [9–15]. Such findings highlights the opportunity to shift the responsibility for harm away from individuals and to instead treat the greater environment as the source of risk and the target for intervention.

Given the dyadic nature of injecting drug partnerships, one aspect of the risk environment that warrants special attention is the role of interpersonal factors, such as closeness and trust. These interpersonal factors help characterize why partners who inject drugs together decide to share equipment, and they can explain the processes by which behaviors become interdependent [16–19]. In previous studies, trust and intimacy have been identified as important facilitators of sharing behavior [11, 20, 21]. Moreover, the frequency of needle/syringe sharing and ancillary injecting equipment sharing is highest among injecting partners with close ties (sexual relationships, family members, and close friends) [22–24]. HCV incidence is also greater among PWID in injecting partnerships that are also sexual relationships, when compared to those in injecting-only partnerships [25, 26]. Such studies reinforce the importance of the social context of injecting drug use and provide impetus for a deeper understanding of how interpersonal dynamics govern sharing behavior. A larger set of findings from studies of heterosexual injecting couples and romantic injecting partnerships has noted the influence of trust, emotional closeness, and gender dynamics on injection risk behaviors [9, 27, 28]. These studies have emphasized the structural and social determinates, and often overlapping, gendered power that accompany sexual injecting partnerships, but they have not yet detailed the interpersonal mechanisms that influence injecting behaviors. To complement the existing research base, studies focused more narrowly on the unit of the dyadic injecting partnership could improve understanding of the interpersonal context within which needles/syringes and ancillary equipment are shared. Further, inclusion of injecting drug partnerships of all relationship types (not just sexual) is needed. Such studies could inform improved measurement of the interpersonal factors inherent in all types of injecting partnerships, and could guide public health approaches that target the dyad, rather than the individual, as the site of risk management [29–31].

Using a qualitative research approach, we sought to examine the interpersonal processes related to drug using behaviors, with a focus on injecting equipment sharing behaviors, among injecting partners. Our study is grounded in a dyadic framework based on Interdependence Theory [32, 33] and addresses two research questions: First, what are the key interpersonal dynamics at play when injecting partners use drugs together? Second, how do such interpersonal dynamics influence needle/syringe and ancillary injecting equipment sharing? Our intention is to offer definitions of key interpersonal factors and detail the various ways these interpersonal factors interact to influence risk and protective behaviors within injecting partnerships. We hope findings can inform epidemiological research through improved conceptualization of the interpersonal dimension of injection drug use.

## Methods

### Theoretical framework

This study was guided by interdependence theory [32, 33], a social exchange theory that focuses on understanding the interaction between partners in relationships to identify the processes dyad members take to balance costs and benefits to the individual and the partnership. Interdependence theory is particularly relevant to understanding needle/syringe and ancillary injecting equipment sharing due to its focus on each partner’s influence on the other’s behavior. Needle/syringe and ancillary injecting equipment sharing are intrinsically dyadic behaviors; a behavioral exchange between two members. Interpersonal dynamics are the affective, normative, and cognitive interactions between two, or more, people [34] and can be conceptualized as the product of their interaction. Interpersonal dynamics are important when considering the risk contexts that influence needle/syringe and ancillary injecting equipment sharing because they affect the ability of one or both partners to coordinate behaviors. A key facet of interdependence theory is the concept of transformation of motivation. Transformation of motivation involves a shift in priorities where dyad members begin to prioritize needs of the relationship over needs of the individual [33]. For this study, the concept of transformation of motivation may explain how behaviors evolve from self-centered to partnership-centered when injecting partners consider the implications of drug-related risks.

### Sample and data collection

Data analysis overlapped with data collection and occurred in an iterative fashion [35]. Between January and April 2013 we conducted one-on-one, semi-structured interviews with young people who inject drugs who reported injecting with another person in the same physical space ≥3 times in past month, purposively sampled from a larger prospective observational study of drug use (participants aged <30 at time of enrollment), the UFO study [36]. Drawing from a list of eligible participants (i.e., participant noted injecting with another person in the past month at their UFO study interview), over the course of 3 months we contacted 30 participants reflecting a diverse representation of gender, injecting behaviors (frequency, drug type, sharing) and number of injecting partnerships; three participants declined due to lack of transportation to the study site. In most cases interviews took place with one member of injecting partnership, although in-depth interviews centered on interpersonal dynamics experienced during recent injecting events with injecting partnerships. The second author (EA), a white/Caucasian female in her late twenties, conducted all interviews. She identified herself as a staff member of the UFO Study and the University of California San Francisco. The interview guide included a series of open-ended questions about recent injecting events with several injecting partners (S1 Table), with an opportunity for participants to note a main injecting partner as “someone they primarily inject with”. Interview discussions focused on events in which high-risk injecting behaviors occurred. Probes were used to elicit information about interpersonal factors (e.g., “What is it that makes people be able to negotiate safer injecting behaviors with some people they inject with but not others?”). Interviews concluded with a series of questions asking participants to identify similarities and differences in factors and situations influencing injecting behaviors across their different injecting partnerships. Interview narratives allowed participants to describe injecting behaviors within multiple partnerships, and probes were used for an in-depth description about interpersonal contexts in which high-risk injecting behaviors occurred. Interviews (lasting between 45 and 80 minutes) were conducted in a private interview room at our field site in downtown San Francisco, CA, and were audio recorded for later verbatim transcription. Participants provided written consent and were provided USD 30 cash remuneration for their time. All research protocols were reviewed and approved by the University of California San Francisco Institutional Review Board. For confidentiality, names of participants have been changed, and all location names have been removed. The participants represented in this manuscript have given written informed consent to publish these narrative details.

### Analyses

Participant interview data reflecting their membership within injecting partnerships were analyzed using the constant comparative approach of grounded theory [35, 37, 38]. Transcripts were initially read in detail, and emerging themes were identified, some of which were specifically explored in subsequent interviews with different partnerships using new interview probes. Weekly analytic sessions during which authors (MM and EA) read and hand-coded field notes and interview data were then summarized, producing categories and relationships among categories used to generate a preliminary set of injecting-specific interpersonal factors. This initial set of factors was further refined through additional in-depth interviews focusing on injecting relationship development and injecting behaviors, sampling different types of partnerships (e.g., HCV serodiscordant, same-gender) and for participants discussing multiple partnerships we compared coded text across partnerships. The transcripts were repeatedly examined and additional interviews conducted to provide ongoing comparisons across the data, allowing for the development of a coding scheme for thematic classification of the data. This process also allowed for a deeper understanding of the nuanced operation of the factors underlying the injecting behaviors within partnerships. Data collection continued until saturation of themes was achieved. Once the coding scheme was completed, the entire set of interview transcripts was analyzed with the goal of raising the analytic level from the categorical to the conceptual [38].

**Fig 1:**
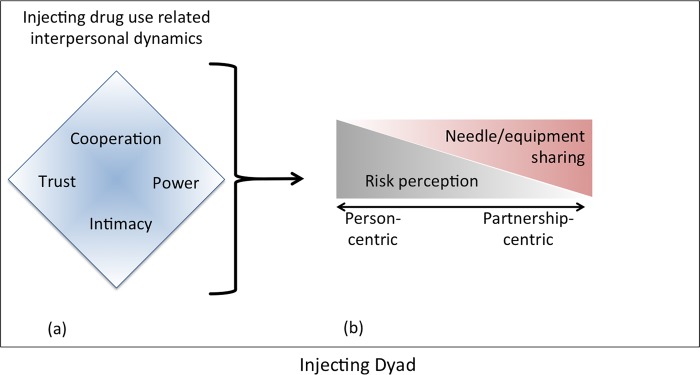
A Conceptual model to represent dyadic injecting drug-related interpersonal dynamics. (a) As injecting partners inject drugs together, their interactions result in injecting-related trust, cooperation, power, and intimacy. These factors interrelate to (b) shape the partners' injecting behaviors according to the judgments they make about protecting themselves and protecting the partnership. The relationship between trust, cooperation, power, and intimacy, on the one hand, with risk perception on the other, is dynamic over time and there is a fluctuation between judgments that protect oneself (person-centered) and judgments that protect the partnership (partnership-centered).

This final level of analysis involved elaborating the relationships among the concepts and identifying a conceptual model representative of the data. We examined our emerging model in the context interdependence theory and considered how our findings elaborated these theoretical constructs. The final conceptual framework for the dyadic injecting drug-related interpersonal dynamics, presented in the results section and Fig 1, is based on our analytic process. Throughout pseudonyms are used to protect participants’ identity.

## Results

### Participant characteristics

Of the 27 interviews, 13 participants were male, and 13 were female; one participant identified as male-to-female transgender. The 27 individual interviews reported in 34 distinct injecting partnerships. The majority identified as White/Caucasian (66%) with an average age of 28 years (21–32); about half (58%) had completed high school. The majority had been unstably housed in the previous month (87%), and had experienced jail or prison in their lifetime (83%); 20% had served time in jail or prison recently (past three months). Twelve (44%) self-reported ever testing positive for HCV. All participants reported partnerships that experienced a circumstance where a needle/syringe or injecting equipment was shared (either knowingly or unknowingly). Fourteen participants in injecting partnerships (50%) reported recent (past 30 days) needle/syringe sharing, and 27 of those in injecting partnerships (100%) reported recent (past 30 days) ancillary injecting equipment sharing within the partnership (Table 1).

**Table 1:** Participant characteristics and partnership narratives.

	Pseudonym	Age	HCV status	Total number of injecting partners in previous 3 months	Partnership gender composition	Number of days injecting together in past month	Live in same place together in past month	Sexual intercourse in past month	Syringe/ needle sharing	Cooker/ container sharing	Relationship type*
Participant	Tom	30	Negative	4	MM	30	Y	Y	Never	Sometimes	Regular sex partner, friend, injecting partner
Partner	John										
Partnership Narrative	John is Tom's main IP. They have known each other for about 3 years and they have been injecting together since they first met. Tom describes the relationship as "co-dependent." They inject heroin together every day. They always inject in the participant’s apartment. They both provide the drugs though participant reports that he has been providing them more frequently at the time of the interview (though he says John will pay him back.) He doesn’t know the other people John injects with though John does know the people Tom injects with. As he describes it John holds relationship power over him (that he acts submissively) and this can translate into injecting power. In general Tom doesn't share works with John because he doesn't know about his injecting practices with his other IPs. But he reports two occasions of having used John's needle after him (the reverse has never happened). Both these situations were due to lack of works. In both situations John injected first because "he was being greedy. "He says he would not share with anyone else.										
Participant	Lulu	29	Negative	19	FM	4	Y	Y	Sometimes	Always	Regular sex partner, friend, injecting partner
Partner	Weasel										
Partnership Narrative	Lulu and Weasel have known each other for 8 years. Been injecting together for 7 years but their sexual relationship only started 2.5years ago. Lulu injects herself in the neck and Weasel in the clavicle. Neither of them have any other spots left they can inject. Weasel can’t inject himself. Lulu always has to use a totally new needle or else she can’t inject herself so when they do share she goes first. Weasel however is the one who usually get the drugs or works to get the money. Lulu has plenty of hook ups too. Lulu sometimes shares needles with Weasel. She reports that he is the only person she has ever shared a needle with and thinks the same goes for him (that he has only ever shared with her). Lulu's very clear that she wouldn’t share with anyone else, even a close friend she had known for a long time.										
Participant	Felix	23	Negative	12	MM	1	Y	N	Sometimes	Rarely	Injecting partner, someone who gives me money, food, a place to stay,
Partner	Lois										
Partnership Narrative	Felix and Lois have an injecting relationship that is largely focused on sex. They've known each other for 2.5years, have been injecting together for 1.5yrs. They inject meth together as a means to stay awake/get turned on. Felix describes the relationship as somewhat coercive, in that Lois will inject him as he's falling asleep in order to initiate sex. Lois is an older man who Felix says purposefully goes to Larkin street to pick up young guys. He has relied on Lois for a place to stay in the past. Felix says that when he first met Lois there was four months when he lived with Lois and was like his “sex slave”–however he was still paying Lois' rent. And Felix explained that Lois didn’t like him to go out because he didn’t want him to bring back other sexy men to his house. While these injecting partners have not injected in the last 30 days, they injected 4 times a day 2 times a month in the 3 months prior.										
Participant	Debby	25	Positive	6	FM	30	Y	Y	Never	Always	Regular sex partner, friend, injecting partner,
Partner	Adam										
Partnership Narrative	Debby and Adam are in a relationship on and off for 7 years. They've known each other for 8 years, have been injecting together for 5yrs. At the time of the interview they'd been together for a year without a break. They inject with each other every day. Debby really trusts Adam because they have known each other "forever" and she knows "everything about him". They split the cost of buying drugs but don't count the pennies. Adam buys the drugs because Debby feels like it’s safer for him to do so (because of her gender). Debby is the one who mixes up the drugs and she always shoots him up. Because she does all this she says she gets to decide who goes first and that if Adam asks her she will inject him first—though not first thing in the morning. If Adam feels too impatient to wait for her, he will waterline into his nose. They mix their drugs in the same cooker but then they pull it up into a clean needle and piggyback half into another clean needle. Debby said she would be willing to do Adam's rinse or share water, things she would never do with anyone else.										
Participant	Bethany	25	Negative	3	FM	30	Y	Y	Never	Rarely	Regular sex partner
Partner	Chris										
Partnership Narrative	Bethany and Chris have known each other for 2 years; have been injecting together almost as long and injected together every day in the previous month. They met when in drug rehab, and have been together on-and-off since. Bethany described herself and Chris' "a unit. "We consider ourselves as one." Bethany said that they were tested for HIV/HCV when in rehab together so "we knew we didn't have anything. And we shared [needles] until last September when Chris tested positive for HIV." Since then they write their names on their needles, and inject in separate areas of the room and dispose of their needles in separate sharps containers.										
Participant	Dominic	22	Negative	12	MF	10	Y	Y	Always	Always	Regular sex partner, friend
Partner	Tiffany										
Partnership 1 Narrative	Dominic has known Tiffany for 4 months, have been injecting together for 2months and injected about 10 times together in the past month. When they first met Tiffany was admitted to the psych ward the following day and called him saying she was scared and could he pick her up. By reaching out to Dominic, Tiffany made him feel as though he was special and not just “a random guy". Dominic reports always sharing a needle with Tiffany (they do speed which they mix in the needle). The first time he injected together, he did Tiffany first and then himself–all with the same needle. Then they did a second round using the same needle.										
Partner	Alicia				MF	1	N	Y	Sometimes	Always	Casual sex partner, injecting partner
Partnership 2 Narrative	Dominic and Alicia met on the street and Alicia invited him to her place where they had sex and injected drugs. Dominic and Alicia did goofballs together one night using her old needle. He specifies that he “wound up taking some of her blood into [him].”										
Participant	Jorge	27	Negative	2	MM	2	N	N	Always	Always	Friend, injecting partner
Partner	Rudy										
Partnership Narrative	Jorge injects only with two people who are a couple, his main IP is the guy, Rudy. They've known each other for 4years, have been injecting together for 1.5yrs. They've injected together twice in the past month. His main IP, Rudy, controls the injecting process from buying to mixing to injecting though they all contribute money to buy. Jorge says that usually they inject when they are partying (drinking) so getting clean needles isn't a priority, but as far as Jorge knows they use a clean needle. They use the same syringe but Jorge always goes first (Rudy injects him). And never borrows, always gives syringes; Rudy prepares the drug; Rudy gets the equipment and drugs. And Rudy is hepatitis C positive, but Jorge doesn't know Rudy's status.										
Participant	Gregory	28	Negative	1	MM	2	N	N	Never	Rarely	Friend
Partner	Harris										
Partnership Narrative	Gregory says he never shares except one time when he was high on meth. Gregory has only ever injected with this one IP, Harris, but Gregory injects alone more often. They've known each other for 8 years, have been injecting together for about 1.5yrs. They have injected together twice in the past month. Gregory reports never borrowing or sharing syringe/needles with Harris; they both prepare the drugs when they inject together; and contribute injecting equipment and drugs equally.										
Participant	Irene	26	Positive	4	FM	3	N	N	Never	Often	Friend, injecting partner
Partner	Kevin										
Partnership Narrative	Irene injects with Kevin because “he is a friend” of hers and she “likes to help him not be sick [withdrawal]”. Irene knows Kevin would take care of her if she overdosed. They've known each other for 7 years, and have been injecting together for 7 yrs. They've injected together three times in the past month. They both prepare the drugs when they inject together; and Irene always provided the injecting equipment and drugs.										
Participant	Mike	30	Negative	5	MF	30	Y	Y	Rarely	Often	Regular sex partner, injecting partner
Partner	Naomi										
Partnership Narrative	Naomi is Mike's main IP. Mike says they've known each other for 9 years and injecting together since meeting. They inject together every day. Mike reports that they cook up their heroin in the same cooker but they use new needles to pull it up and then piggyback into two other new needle/syringes, which they use to inject. It is easy for them to get new equipment so they always have some with them. Mike does report they sometimes run out of new equipment, in which case they reuse needles/syringes. But they keep needles/syringes in separate places and sometimes mark them with a marker. Mike didn't go into detail about how they inject if they are reusing their equipment. Mike also says there are things he does with Naomi that would never do with anyone else, like using the same needle multiple times to pull up from a cooker. They rarely share/borrow; often prepare in same container. Mike always prepares the drugs, but both equally provide new equipment and drugs when injecting together.										
Participant	Olivia	24	Positive	3	FF	5	Y	N	Never	Often	Injecting partner, roommate
Partner	Prank										
Partnership Narrative	Olivia injects with her roommate, Prank, because they live together. They've known each other for 2 months and have always injected together. They've injected together 5 times in the past month. They are each other's look out—they live in a youth center. Olivia usually provides the drugs, equipment and prepares the drug. They never borrow or share needles/syringes, but often mix up in same container, and reuse their container.										
Participant	Russell	29	Positive	2	MF	3	N	N	Never	Sometimes	Acquaintance, injecting partner
Partner	Queenie										
Partnership Narrative	Russell's main IP is an older woman, Queenie, who lives in the same building as a friend. Russell has known her for about 4 months and have injected together for the same amount of time. They injected together three times in the past month. Russell injects with Queenie because she has a good connection for heroin. Queenie is HIV+. Russell usually buys the drugs and they split them 50/50. Queenie provides the cookers and they each provide needles/syringes for each other (because Russell likes shorts but can get longs more easily and vice versa for Queenie). Russell always asks Queenie if the cookers/needles are clean and inspects them for signs of previous use. They sometimes mixed up in same container.										
Participant	Sophia	29	Positive	12	FM	25	Y	Y	Rarely	Always	Regular sex partner, friend, injecting partner, someone who gives me money, food, or a place to stay
Partner	Arthur										
Partnership Narrative	Sophia's main IP is also her boyfriend, Arthur, and they inject together almost every day (25/30 days). They have known each other and injected together for 2.5years. They rarely share needles because she is a "stickler" (while Arthur IP is not). Sophia is adamant that she would never have to share in San Francisco because of the number of places to get new needles/syringes. Sophia and Arthur have a complicated procedure for injecting because Arthur cooks up the heroin, Sophia mixes the speed and then she piggybacks into his needle some speed and take heroin for the cooker he used. They always mix in same container. And sometimes reuse their needles/syringes.										
Participant	Victor	27	Negative	2	MM	3	N	N	Rarely	Rarely	Acquaintance,
Partner	Ron										
Partnership Narrative	Ron is an acquaintance of Victor's but Victor normally injects alone. They've injected together 3 times in the past month. They've known each other for 6 months, and have been injecting together for 3 months. Recently when they injected together, Victor’s syringe was blocked so he ended up using Ron’s already used needle/syringe, even though he didn’t want to at first–he felt manipulated into it (because they were trading pills for heroin and Ron had already taken the pills) and was very upset/angry. He hasn’t seen Ron since.										
Participant	Yvonne	32	Negative	150	FM	30	Y	Y	Rarely	Sometimes	Regular sex partner, injecting partner, "street husband"
Partner	Zack										
Partnership Narrative	Yvonne and Zack inject together every day. They've known each other for 4 years, have been injecting together for 3 years. When Yvonne injects with Zack, she cooks up the drugs, pulls it into one syringe and then backloads the other two (which are new). If they are running low on needles they know they'll reuse their needles/syringes at some point, so Zack ties a different color tourniquet around his needles. Yvonne never uses his needle, but Zack does use Yvonne's sometimes. They always mix up in the same container.										
Participant	Alexa	26	Positive	100	FM	30	Y	Y	Never	Always	Regular sex partner, "husband"
Partner	Brian										
Partnership Narrative	Alexa and Brian have known each other and injected together for 2 years, and injected together daily in the previous month. Their relationship is described as tumultuous. At the time of this interview they were "on the outs" because Alexa had just kicked Brian out the previous night. Alexa gets all their drugs, prepares them and inject both Brian and Alexa, and she does him first because "otherwise he’ll complain". But if it’s later in the day she’ll inject herself first. She said that the fact that she buys the drugs makes it easier for her to decide how she injects.										
Participant	Cecil	29	Positive	4	MM	2	Y	N	Rarely	Rarely	Friend,
Partner	Jim										
Partnership Narrative	Cecil and Jim are close friends but they haven’t known each other for a very long time. They've injected together twice in the past month. It’s a new friendship but Cecil feels close to him because of their shared experiences. They don't usually share anything but there have been occasions of sharing a cooker (in which case Cecil injected first), and of Cecil are sharing his needle/syringe with Jim. Cecil does accept preloaded needles from Jim because "he trusts him" and he's his best friend.										
Participant	Don	30	Negative	50	MM	10	N	N	Never	Rarely	Acquaintance, injecting partner
Partner	Simon										
Partnership Narrative	Don and Simon are friends and they never share needles/syringes, and rarely mix up in the same container. They've known each other and injected together for 4mnths. They've injected together 10 times this month. Its a newer partnership for Don and every time they inject together the sequence/context is different, regarding drug preparation										
Participant	Megan	31	Negative	6	FF	2	Y	Y	Never	Always	Regular sex partner, friend, injecting partner
Partner	Gabby										
Partnership Narrative	Gabby is Megan's girlfriend. They've known each other for 8 years, have been injecting together for 5mnths They have injected together 2 days this month. Megan is in charge of getting the drugs because she makes more money. They never share needles/syringes, but always mix drugs up in the same container. They often reuse their own needles/syringes. Normally they inject together 8 xs per month but this month they broke up for a while.										
Participant	Kiki	28	Positive	30	FM	15	Y	Y	Sometimes	Always	Regular sex partner
Partner	Cesar										
Partnership Narrative	Kiki and Cesar have known each other for 2 years, injected together almost all that time and injected together 15 days during the previous month. They normally inject everyday together, but Cesar was in jail at the time of interview. When the inject together they inject each other sometimes, and sometimes inject him/her self. Cesar usually inject himself first because he has been injecting longer, and "its harder to find a vein". Cesar usually prepares the drugs in a previously used container. Cesar has HCV, so they try not to share needles/syringes but have when out of clean needle/syringe.										
Participant	François	26	Negative	10	MM	20	Y	N	Rarely	Rarely	Friend, injecting partner
Partner	Eduard										
Partnership Narrative	Francois and Eduard are roommates/acquaintances. Eduard usually mixes and cooks up the heroin. Eduard would inject himself first if Francois needed help injecting; Francois injects in the groin. Francois doesn’t say anything about having to wait to inject because he didn’t think Eduard would listen. They've known each other and been injecting together for 6 months. Francois rarely borrows Eduard's needles/syringes and never shares his. They rarely share containers since they mix their drugs up in separate containers.										
Participant	Blanca	24	Negative	4	TT	2	Y	Y	Never	Rarely	Regular sex partner, "girlfriend"
Partner	Maria-Luisa										
Partnership Narrative	Blanca and Maria-Luisa are in a relationship. They've known each other for 2years, and have been injecting together for 8 months. They are both transgender. Blanca describes herself as a control freak, even with Maria-Luisa. Blanca insists that she and Maria-Luisa not share because she won’t share with anyone. Blanca says Maria-Luisa respects Blanca for that. Blanca is open to the possibility of sharing a needle with Maria-Luisa, which is why it’s so important that she doesn’t share with other people.										
Participant	Miguel	22	Negative	40	MF	30	Y	Y	Often	Always	Regular sex partner, "girlfriend"
Partner	Gina										
Partnership Narrative	Miguel and his girlfriend Gina used to inject together and got clean together. They've known each other for 7 years, have been injecting together for 2 years. Miguel always injects her because she doesn't know how to do it herself and doesn't want to learn (because she didn’t want to be able to inject at will). Miguel and Gina always shared NS. They would take turns going first. They would use one needle for only one round (i.e. once on each of them). Miguel said that the only time he shared with anyone else was when Miguel was when he didn’t have a needle, or one time when the one he was going to use broke. Miguel cleaned out the other persons needle with bleach and water before using it but he knows that doesn’t kill everything and he was really worried for a few weeks after that. They always shared containers.										
Participant	Reid	28	Negative	10	MM	20	Y	N	Sometimes	Often	Friend, injecting partner
Partner	Dan										
Partnership 1 Narrative	Reid and Dan have known each other and been injecting together for 7 years. Usually Reid cooks up the drugs and then piggy backs it from a clean needle, which he uses to inject himself, into Dan's needle. Reid thinks this could put him at some risk because his needle probably touches the inside of Dan's needle and even sometimes the fluid rises up to touch it and he doesn’t know if Dan is reusing needles or not. When question as whether Reid would inject like that with someone else he said no. Dan uses Reid's needles after him even though Reid has told him he has had a blood infection. Reid doesn’t know if he might also use other people’s needles. They often share containers when mixing up drugs.										
Partner	Liz				MF	1	N	Y	Sometimes	Often	Casual sex partner
Partnership 2 Narrative	Reid and Liz are boyfriend and girlfriend. They've known each other for 7 years, have been injecting together for 5 years. Reid sometimes uses her NS after bleaching it but Liz would never use Reid’s They often prepare drugs in the same container that is being reused. Reid prepares the drugs and provides injecting equipment and drugs.										
Participant	Anne	21	Positive	20	FF	15	Y	N	Never	Sometimes	Friend, someone who gives me money, food, or a place to stay
Partner	Becca										
Partnership 1 Narrative	Since becoming homeless, Anne relies on Becca for a place to stay. They have known each other for 1.5 years and have been injecting together the same amount of time. Anne has injected most often with Becca over the previous 3 months. Anne buys Becca's drugs from Becca and she injects Anne. Anne worries that Becca will make a mistake and pick up the wrong needle when they inject together since Becca often dozes off when injecting Anne. They sometimes reuse containers to prepare drugs.										
Partner	Henry				FM	21	Y	Y	Sometimes	Always	Casual sex partner, injecting partner
Partnership 2 Narrative	Anne met Henry through some friends about a month before the interview, and they've been injecting for that 1-month together. Henry lives in Seattle and is visiting San Francisco. Anne describes the relationship as "almost immediately a boyfriend-girlfriend relationship", though short-lived since Henry was only in SF for 3 weeks. They injected 3 times a day, during the 3 weeks together. Anne and Henry asked each other before sharing a needle whether or not either of them “had something.” This, however, as she says, was just “all by word.” Once they had shared a needle one time, Anne said, "it wasn’t as big a deal to share again". They always mixed their drugs up in the same container or reused containers.										

* from survey question: How would you define your relationship with [partner] (mark all that apply): regular sex partner, casual sex partner, friend, sibling/relative, dealer, acquaintance, someone I inject with, someone who gives me money, food, or a place to stay, other (specify).

Abbreviations: HCV, hepatitis C virus; IP, injecting partner; NS, needle/syringe; Y, Yes; N, No; F, female; M, Male; SF, San Francisco.

### Key interpersonal factors underlying how injecting partners use drugs together

Four interpersonal factors dominated the narratives: trust, cooperation, intimacy, and power. Briefly, we define and describe each factor. “Quoted” text indicates terms or statements cited during participant interviews.

#### Trust

Trust in the context of an injecting partnership often translated to deliberately not doing things to hurt each other, articulated as *“not fucking each other over*.*”* Trust was sometimes expressed as an understanding between partners to *“look out”* for each other’s well-being. For example when asked, *“What does trust mean to you in terms of injecting*?*” François* explained, *“[I]t just means that they’re looking out for my best interest*. *If they had something [HCV/HIV] they wouldn’t want me to use their dirties; they would tell me*. *They would tell me*. *I’d know*. *Because why would they… That would be weird*.*”* Many participants described trust as an extension of expecting their injecting partner to practice good judgment when injecting. For all participants, trust was based on an expectation to treat each other with a mutual level of respect.

Participants described trust as an impression stemming from previous drug-using behaviors and/or non-drug related interactions that influenced partnership-level trust and how they injected drugs together. *Dominic* explained, “*I trust Tiffany enough to let her put a needle in my arm… I have to also trust her with things like [when] I leave her in the room with my jacket which has important documents in it … I know Tiffany won’t run off with it*.*”* When asked to rank how much they trust their injecting partners on a scale from 0–10, only a few of the participants applied a score of 10. The general sentiment was that *“[Y]ou really can’t trust anybody 100 percent on the streets*.*” Reed* went on to add that he *“never give[s] [his] full trust to anyone… A [score of] Seven means I’ll give them just enough trust to keep them around*, *but not enough trust that will harm me in any sort of way*.*”* Some interviewees observed that one’s own flaws could temper the absolute trust in another. *Debby* explained how she had been dishonest with her husband about using drugs after two years of sobriety, *“[Y]eah…yeah*, *I trust him*. *I really trust him*. *He’s a good guy*. *But I also kind of don’t [trust him] because look what I’m doing [injecting drugs]*, *behind his back*.*”* This feeling of needing to guard oneself was shared by most participants. *Anne* explained that based on an experience with a previous injecting partner *“[I] can’t really trust anyone because everyone is really out for themselves*.*” Debby* was one of a few participants that gave her injecting partner a 10 on the trust scale, since *“[I have] known Adam forever… knows everything about him… seen his test results on paper… no reason to not trust him… he hasn’t done sneaky shit behind my back or anything*.*”*

#### Cooperation

Cooperation surfaced as the unconscious and conscious give and take between members of an injecting partnership when one member’s action benefited their partner and also themselves, either directly or indirectly. Cooperation was related to the extent to which a participant and their injecting partner helped each other out and took care of each other when it came to drugs, injecting, or more generally. Cooperation manifested and deepened as partners helped each other procure drugs, kept each other out of trouble (for example, from police or others within the community or in deterring perpetrators of violence), prevented/responded to overdose, promoted safe injecting practices, assisted injecting, and helped in situations of withdrawal.

Some participants reported that it didn’t really matter which partner more frequently paid for drugs because they shared things equally and took turns making money. There was a long-term vision of *“pay off”* that included resources not limited to drugs (such as food and housing). Drug-related norms (e.g., when/how pool drugs) helped govern expectations for cooperation within injecting partnerships. Participants noted that norms informed by the larger drug-using network influenced expectations within the early stages of injecting partnerships. They also referenced how street norms affected the discussion of HCV status between injecting partners. Mike noted, *“a lot of people that know they’re infected say it right up front*. *And I think that’s part of the street rule…”*

#### Intimacy

Intimacy was described as a *“feeling of closeness*,*”* familiarity, strong connection, or *“caring for”* one’s injecting partner, in addition to physical/sexual attraction for some. Even in non-sexual injecting partnerships, an earlier phase of being *“dope buddies”* could evolve into a deeper emotional partnership.

Many respondents remarked that relationship duration was only part of what drove injecting partnership intimacy. They acknowledged the unique context that is living on the street and/or being removed from family increased feelings of intimacy when injecting. Participants stated they were *“guard[ed]”* against people generally, thus amplifying a feeling of *“intimacy”* or *“connection”* with injecting partners who had many shared experiences together.

A desire for connection caused some to have a high expectation of their relationship or to perceive closeness early in a relationship when the feeling might be a projection of the need for closeness. For example, when *Anne* was asked what made her more likely to share with *Henry*, she notes, “*Probably because I was like yearning for a guy companion for so long*, *and I finally got it*, *so I just wanted to put all my faith and trust in him because of the way he was treating me*, *like really sweet*, *like yeah*, *he was treating me at the moment*, *made me feel like I could trust him*.*” Jorge’s* response to what defines closeness and how it influences injecting behaviors was similar, stating,

“…*Like I guess the…events that I’ve been through with [Rudy]…I cannot name a specific moment*. *All I know is for me the times [sharing equipment] happened…like with my guy friend*, *we were sitting in the space toilet (outdoor toilet) one day*, *and he was trying to do this shot of speed*, *and he kept flashing blood up into [the syringe]*. *And finally*, *I got so frustrated he didn't even want to do it anymore*. *And I was like*, *"I'll hit you*.*" And he was like*, *"No*, *I don't even want it anymore*.*" And he's like*, *"You can do it if you want*.*"[using the needle with his blood in it] And I thought about it*, *and I was like*, *‘Gee*, *I've been through so many things with you already*, *and I've known you for a while*, *and we've basically been living together for the last couple months*. *‘Sure*. *Why the fuck not*?*' you know*, *and I did it*.”

#### Power

Drug-related power may encompass a power ratio within partnerships, can be dynamic over time and may vary with context. Which partner holds more power within a partnership may differ across different drug using situations or as individual resources and opportunities change (e.g., one partner gets an apartment, and that partner is then able to dictate where and how they inject drugs together). For example, *Blanca* moved into a room in a single room occupancy [short-term stay apartment] building and, due to her stable housing status, was able to dictate where and who prepared the drugs when *Blanca* and *Maria-Luisa* injected together. This resulted in *Blanca* taking a second hit using the drug residue from *Marie-Louise's* cotton.

Power dynamics may result in one partner feeling *“fearful”* or uncomfortable speaking up if they are engaging in undesired injecting behavior. Some participants noted that power dynamics were enforced explicitly through violence or verbal abuse, or subtly through guilt or manipulative behaviors. For example, *Henry* was more experienced and older than *Anne*. She went along with what he said. *Henry* would buy the drugs, although *Anne* made all the money. *Anne* felt like there was a time when he was making his shots stronger than hers and would get violent when she suggested so.

*“Henry was definitely more dominant* … *He had a score… [he] had to inject first*, *you know*, *then he would hit [inject] me second*. *Henry liked that I didn't know how to [inject myself] because that made me have to rely on him* … *so [I] couldn’t leave him… He became really like dominant*, *and like controlling*.*”* -*Anne*

### Interpersonal dynamics in the injecting partnership model

These data informed the development of a model of dyadic injecting drug-related interpersonal dynamics. At the core of the model is dyadic risk perception, defined as the balance between protecting oneself and protecting the partnership. Extending from interdependence theory [32], dyadic risk perception in the context of injecting partnerships can exist along a continuum between doing what is in the best interest of the individual (person-centered) and taking selfless actions in the interest of doing what is in the best interest of the partnership (partnership-centered). The connection between interpersonal factors (trust, cooperation, intimacy, power) (Fig 1A) influences the transition from person-centered to partnership-centered risk perception (Fig 1B) and may explain why high-risk injecting behaviors occur more often when trust, cooperation, intimacy, and power are at higher levels. For example, when these interpersonal factors intensify, the perception of risk to self-reduces as part of the transition toward a more partnership-centric perspective, and the likelihood of needle/syringe and ancillary injecting equipment-sharing increases.

Interdependence theory posits that the shift from person-centered to partnership-centered behaviors is the basis of a transformation of motivation [33]. Participant narratives illustrated how risk perception also changes as injecting partners move from person-centered to partnership-centered.

#### Interpersonal dynamics from person-centric narratives

A person-centric decision-making approach often included relying on physical appearance ‘data’ when assessing injecting risk. Participants explained that if someone didn’t know how to take care of him or herself, they questioned how they could care about protecting themselves from diseases. *Sophia* tends to inject with people who have the same principles as her own,

*“…To be honest, most people that I shoot dope with have the same pretty much principles and values as I do when it comes to shooting dope. I have a tendency to—most people that don't care, you can kind of tell…Their outward appearance and the state of their home and shit like that. You can totally tell who is more likely to stick to fuckin’ using clean works and who’s like, I don't give a fuck*.*”*

*Reid* explains that when they shared a cooker, he would pull up first because he knows that he *“has nothing”* while *Dan “looked kind of like the guy that you wouldn’t want to shoot up after*.*”* In a separate incident with a different injecting partner, *Reid* explains that he used his intuition again to determine that *Dan* was not using a dirty cooker and therefore shared the cooker when injecting together.

*Cecil* described his own manipulative behavior with a partner, *Jim*. *Cecil* had introduced *Jim* to injecting drugs, and he would pressure *Jim* to inject with him even when he didn’t want to so he would have someone to inject with and nod off with.

*"… there were times I would have something made up, and I would be like, Jim, you want this? And he would be like, no, not really. And I’d be like, you sure you don't want this. Peer pressure. And then sometimes it would get to him. I’d be like, come on, just shoot this up so we can sit here and be nodding out together… Just go on and shoot it up… Millions of different things, I would say, you know. I used to be really manipulative*.*”*

#### Examples of interaction of factors leading to a shift to partnership-centric risk perception

Participants remarked that drug-related cooperation was a major factor in developing trust and was quick to add how the harsh conditions of street life elevated the role of cooperation between injecting partners beyond how they used drugs together. Even though *Cecil* scored his trust in his injecting partner at a 4 (out of 10) *“Jim would take a bullet for me*, *and in return*, *I would take a bullet for him*, *you know*.*”* Suggesting that even if absolute trust doesn't exist, partners were willing to increase their personal risk to serve the partner’s needs or the needs of the partnership.

However, most people in this study also felt that this connection was fragile. Some participants described feelings of trust becoming amplified through feelings of loneliness. When asked to account for why they shared with a long-time injecting partner, they spoke of trusting that partner that influenced their decision to share needles/syringes or injecting-related equipment.

*“a lot of people—especially lonely people—and there’s a lot of lonely injectors out there; they get confused with the closeness*. *And they confuse that closeness into care and all this other stuff*. *That’s why they may feel comfortable*.*”* -Yvonne.

*Anne* also talked about how *Henry* may have feigned intimacy to manipulate her to provide him with drugs, "*I don’t know if it was just a mind game*, *like to get him to—get me to get close to him or something*.”

Sharing injection equipment was seen as part of one’s larger relationship context. *“I didn’t mind sharing needles at all with Naomi*. *[I] Shared everything else with her*, *so…” -Mike*. Even for those who did not share any needles/syringes or injecting equipment, the possibility of stretching those boundaries was only considered with someone in this special role. If it came down to necessity *Debby* says she would share with *Adam* but only him “*because Adam’s not just my sexual partner…But he's my best friend*, *too*. *So*, *you know*, *and we use together all the time*. *So*, *I don't know*, *just because we share everything*. *We share food; we share cigarettes; we share drugs*.”

*Mike* notes the role trust can play in the shift from person-centric to partnership-centric risk perception:

*"… [h]onestly the more trust you have with people you use with, I think, puts you more at risk. At least for me, because I know the more I trust somebody, the more eventually I'm more and more prone to be like, "Yeah, sure. Fine, why not? I'll use one of your dirties*.*"*

*Lulu* and *Weasel* have been injecting together for seven years and were in a sexual relationship for the previous two years. Each has other injecting partners, although according to *Lulu*, they won’t inject without each other present–to do so would be paramount to cheating. *Lulu* explained a situation representing partnership-centric risk perception. *Lulu* usually buys the drugs when they inject together, and “*Lulu injects Weasel because he can’t do it [himself] and sometimes Weasel will inject her [Lulu]*.” If there is only one new needle, *Weasel* will let *Lulu* inject herself first since she can’t inject herself in the neck with a dull needle. *Lulu* reports never having shared with anyone else. Whether conscious or unconscious, *Lulu* put increased her personal risk for HCV to cater to Weasel's injecting needs.

For some, as trust, intimacy, and power developed over the course of the injecting partnership, the influence of drug-related norms became weaker. Instead, an injecting partnership’s cooperation became more nuanced and reflective of the unique injecting partnership. For one partnership, interpersonal dynamics contributed to simultaneous person-centric and partnership-centric risk perception during an injecting event. *Miguel* explained that he felt responsible for protecting his girlfriend and injecting partner, Gina since she began using because of him. By providing *Gina* drugs, *Miguel* ensured his girlfriend did not have to look elsewhere to get drugs. *Miguel* felt that this protected her from having to sell her body for drugs. By protecting *Gina*, he eased his guilt of having introduced her to injecting drugs. *Miguel* also benefitted from this arrangement and because he preferred to inject alone or just with his girlfriend since he did not want to interact or share drugs with other people.

Participants noted that interpersonal factors (trust, cooperation, intimacy, power) changed over time. Therefore, changes in interpersonal dynamics resulted in a shift back and forth between the person-centric risk perception and partnership-centric risk perception. Changes in trust and power appeared to be responsible for shifts from partnership-centric back to person-centric risk perception. A reduction in trust could develop from unclear motivations of an injecting partner or from witnessing “selfishness” or other self-serving behavior. One time *Olivia* and *Priyanka* who had previously told *Olivia* she had HCV, dropped their needles on the floor. Priyanka told *Olivia* it was fine, she didn't have HCV anymore, and they just picked up the previously used needles, prepared their shot, and injected with the reused needles. After the event, *Olivia* was *“really pissed off because (s)he felt like her friend had lied and put me at risk*.*” Olivia*’s decision to not use drugs with Priyanka any longer represents a shift back toward person-centric risk perception (Fig 1B).

A similar shift from partnership-centric back to person-centric risk perception occurred with *Alexa’s* partnership with *Brian*. *Alexa* related that she had previously trusted *Brian* but her trust was reduced due to his unwillingness to reciprocate: *“Brian just doesn’t care and doesn’t want to throw his own weight [contribute equally to the partnership]*. *He’s being very selfish*, *just taking advantage of everything and anyone he can*. *Including me*.*”*

## Discussion

We investigated the ways in which interpersonal factors influence drug-using behaviors, with a focus on needle/syringe and ancillary injecting equipment sharing behaviors, in injecting partnerships. First, the findings point to the importance of trust, cooperation, intimacy and power as key interpersonal factors underlying how injecting partners use drugs together. Second, the interaction of these factors with each other has a collective influence on the type (person-centric vs. partner-centric) and level of risk perceived and enacted by injecting partners. Our findings, summarized through an expanded conceptual model, highlight the influence of interpersonal dynamics in all types of injecting partnerships—not only those in close or romantic relationships.

The finding that trust and intimacy play pivotal roles in the shift from person-centric to partnership-centric risk perception shared experiences and external threats to the injecting partnership, reinforcing the need to protect the partnership. Third, absolute trust; individuals may be willing to increase one's own personal risk to serve the needs of the injecting partnership. Previous work has similarly acknowledged the roles of trust and intimacy as facilitators of needle/syringe and ancillary injecting equipment sharing among partners in close relationships [11, 21, 39], and our findings. Rhodes et al. have presented a risk environment framework for how risk is conceptualized by individuals and romantic couples, and Rance et al. expanded to acknowledge negotiated safety in response to perceived risks.[40] Underlying these two frameworks is the recognition that risk incorporates more than just viral risk; individuals and couples make decisions about behaviors such as injection drug use based on complex, and at times competing risks that change over time and across contexts. Our findings of the impact of injecting-related trust and intimacy reinforce the idea that both risk conceptualization and risk-related decisions among partners who inject drugs are complex, dyad context-dependent, and take into account more than just viral risk; public health interventions for PWID must consider this complexity to be effective.

We also found that cooperation and power each uniquely impact how injecting partners use drugs together. The balance of injecting-related power may fluctuate depending on different drug-using situations or as individual material, or social resources change. While previous studies have focused on the role of sexual power on injecting behaviors within sexual partnerships [41, 42], our study findings identify injecting-related power in the context of all types of injecting partnerships—not only sexual relationships. Moreover our finding that many interpersonal factors besides power (trust, cooperation, intimacy) play an important role in the way injecting partners perceive risk may explain the null association between the sexual relationship power scale, a quantitative measure of sexual power, and sharing behaviors among a similar sample of injecting partnerships [43]. Cooperation was closely tied to reciprocity related to procuring drugs, preventing overdose and withdrawal, and assisting injecting. Instrumental resources (i.e., money, housing, drugs) sharing as a form of cooperation has produced mixed results in quantitative studies of needle/syringe and ancillary equipment sharing [44, 45], and has been posited as a source of asymmetrical power relations producing heightened injecting risk behaviors in injecting partnerships dissimilar in gender or age [46]. Our findings suggest that considering the role cooperation and power within partnership’s risk-perception type (person-centric or partnership-centric risk perception) could support interpretation of future studies of needle/syringe and ancillary equipment sharing. Further, they again reinforce the need to look beyond viral risk in partnerships with partnership-centric risk perception qualities and recognize that the care and stability that accompanies such partnerships may reduce risk in other areas of their lives.

Lastly, our adapted conceptual model illustrates the utility of interdependence theory for examining interpersonal dynamics in injecting partnerships. Interdependence theory emphasizes understanding the outcomes that partners experience by analyzing how the two partners interact and influence each other dynamically. The connection between injecting-related trust, cooperation, intimacy, and power reflect the continuous, bidirectional influence that occurs between two injecting partners. Interdependence theory’s core tenet is that transformation of motivation serves as the basis for the shift from person-centered to partnership-centered behaviors [33]. Our findings suggest that while interpersonal factors levels change and risk perception may shift, increased perception of risk may not translate to a reduction of risky behavior. One interpretation is that trust acts as a short-cut for risk decision-making. Trust may signal a sense of normalcy in routine activities, like *Tom* always preparing his shot second from the same container after *John* does, without questioning whether the behavior is safe. It is when trust breaks down that safety assumptions may be challenged, and risk re-assessment may be required. A shift between judgments that protect self (person-centered) and decisions that protect the partnership (partnership-centered) may help to explain or account for why needle/syringe and equipment sharing practices occur more frequently in relationships with closer ties. However, the subjective nature of trust and intimacy may complicate approaches to developing prevention strategies. In contrast, cooperation and power were often demonstrated in more measurable ways (e.g., verbal/physical abuse or exchange of material goods) in our study. One potential approach for prevention strategies could be to leverage this information to encourage injecting partners to promote a balance in interpersonal factors rather than target a single factor.

To date most applications of interdependence theory have focused on heterosexual married couples and more recently, gay male couples, to hypothesize how interpersonal or relational factors influence health behavior [47–51]. To our knowledge, this is the first study to apply interdependence theory to drug-using dyads. This work also borrows from others in the field of relationship research that recognize that partners in marginalized groups, such as gay couples or injecting partners, who lack social network support from traditional sources (e.g., friends, family) and engage in socially stigmatized behavior, may exert a stronger influence on each other’s behavior compared to partners in less marginalized groups [52]. Partnership-focused prevention strategies targeting trust, cooperation, intimacy, and power, specifically promoting a balance between partners, may help reduce risky injecting practices while retaining relationship ties. For example, interventions to improve communication skills have helped improve condom use negotiation among sexual couples and may offer an area for development to reduce needle/syringe and ancillary equipment sharing among injecting partners [53].

We note several limitations. We lacked data from both members of the injecting partnerships (dyadic data), limiting our comparison of interpersonal factor levels across partnerships. We also lacked longitudinal data, instead relying on historical accounts, which may be subject to recall bias. We chose to focus study findings on interpersonal factors, bound by the partnership unit rather than the partnership’s broader physical environment, to deepen the field's understanding of the role of interpersonal dynamics on injecting behaviors. The purpose of our model is three-fold. First, our findings are situated within an epidemiologic framework aimed at identifying interpersonal factors among drug injecting partnerships that can be measured and studied in future research. Second, we hope to inspire new approaches to understanding how relationships can reduce harm among people who inject drugs together, including potential avenues for health interventions that improve health and well-being. As our findings are situated within an epidemiologic framework aimed at identifying factors for subsequent measurement, study, and intervention, the proposed model needs empirical validation. We encourage others in the field to examine its application and recommend modifications. It provides a framework for discussion about the nature of injecting partnership relationships and how injecting members interact communally. We emphasize the injecting partnership as a critical environment and unit of study and join others in the field’s call to value the supporting social ties between people who inject drugs [30, 54–56].

## Supporting information

S1 Table Qualitative interview guide. (DOCX)

## References

[1] Aceijas C, Rhodes T. Global estimates of prevalence of HCV infection among injecting drug users. The International journal on drug policy. 2007;18(5):352–8. Epub 2007/09/15. S0955-3959(07)00091-6 [pii] 10.1016/j.drugpo.2007.04.004 . 17854722

[2] Morris MD, Shiboski S, Bruneau J, Hahn JA, Hellard M, Prins M, et al Geographic Differences in Temporal Incidence Trends of Hepatitis C Virus Infection Among People Who Inject Drugs: The InC3 Collaboration. Clin Infect Dis. 2017;64(7):860–9. Epub 2017/04/01. 10.1093/cid/ciw869 . 28362947 PMC5439493

[3] Hagan H, Pouget ER, Des Jarlais DC, Lelutiu-Weinberger C. Meta-regression of hepatitis C virus infection in relation to time since onset of illicit drug injection: the influence of time and place. Am J Epidemiol. 2008;168(10):1099–109. Epub 2008/10/14. 10.1093/aje/kwn237 18849303 PMC2727245

[4] Suryaprasad AG, White JZ, Xu F, Eichler BA, Hamilton J, Patel A, et al Emerging epidemic of hepatitis C virus infections among young nonurban persons who inject drugs in the United States, 2006–2012. Clin Infect Dis. 2014;59(10):1411–9. Epub 2014/08/13. 10.1093/cid/ciu643 . 25114031

[5] Thorpe LE, Ouellet LJ, Hershow R, Bailey SL, Williams IT, Williamson J, et al Risk of hepatitis C virus infection among young adult injection drug users who share injection equipment. Am J Epidemiol. 2002;155(7):645–53. Epub 2002/03/27. 10.1093/aje/155.7.645 . 11914192

[6] Hagan H, Pouget ER, Williams IT, Garfein RL, Strathdee SA, Hudson SM, et al Attribution of hepatitis C virus seroconversion risk in young injection drug users in 5 US cities. The Journal of infectious diseases. 2010;201(3):378–85. Epub 2010/01/08. 10.1086/649783 . 20053137

[7] Gibson DR, Choi KH, Catania JA, Sorensen JL, Kegeles S. Psychosocial predictors of needle sharing among intravenous drug users. The International journal of the addictions. 1993;28(10):973–81. Epub 1993/08/01. . 8407025 10.3109/10826089309062177

[8] Falck RS, Siegal HA, Wang J, Carlson RG. Usefulness of the health belief model in predicting HIV needle risk practices among injection drug users. AIDS education and prevention: official publication of the International Society for AIDS Education. 1995;7(6):523–33. Epub 1995/12/01. . 8924349

[9] El-Bassel N, Strathdee SA. Women Who Use or Inject Drugs: An Action Agenda for Women-Specific, Multilevel, and Combination HIV Prevention and Research. J Acquir Immune Defic Syndr. 2015;69 Suppl 2:S182–90. Epub 2015/05/16. 10.1097/qai.0000000000000628 . 25978486 PMC4932853

[10] Rhodes T. The ‘risk environment’: a framework for understanding and reducing drug-related harm. International Journal of Drug Policy. 2002;13(2):85–94. 10.1016/S0955-3959(02)00007-5.

[11] Rhodes T, Treloar C. The social production of hepatitis C risk among injecting drug users: a qualitative synthesis. Addiction. 2008;103(10):1593–603. Epub 2008/09/30. 10.1111/j.1360-0443.2008.02306.x . 18821870

[12] Janulis P. The micro-social risk environment for injection drug use: An event specific analysis of dyadic, situational, and network predictors of injection risk behavior. The International journal on drug policy. 2016;27:56–64. Epub 2015/11/05. 10.1016/j.drugpo.2015.09.006 26530884 PMC4715965

[13] Rhodes T, Singer M, Bourgois P, Friedman SR, Strathdee SA. The social structural production of HIV risk among injecting drug users. Soc Sci Med. 2005;61(5):1026–44. Epub 2005/06/16. 10.1016/j.socscimed.2004.12.024 . 15955404

[14] Strathdee SA, Shoptaw S, Dyer TP, Quan VM, Aramrattana A. Towards combination HIV prevention for injection drug users: addressing addictophobia, apathy and inattention. Current opinion in HIV and AIDS. 2012;7(4):320–5. Epub 2012/04/14. 10.1097/COH.0b013e32835369ad ; PubMed Central PMCID: PMCPmc3646543. 22498479 PMC3646543

[15] Wilson H, Brener L, Mao L, Treloar C. Perceived discrimination and injecting risk among people who inject drugs attending Needle and Syringe Programmes in Sydney, Australia. Drug Alcohol Depend. 2014;144:274–8. Epub 2014/09/23. 10.1016/j.drugalcdep.2014.08.018 . 25236889

[16] Gyarmathy VA, Li N, Tobin KE, Hoffman IF, Sokolov N, Levchenko J, et al Injecting equipment sharing in Russian drug injecting dyads. AIDS and behavior. 2010;14(1):141–51. Epub 2009/02/14. 10.1007/s10461-008-9518-6 19214731 PMC2818991

[17] Morris MD, Bates A, Andrew E, Hahn J, Page K, Maher L. More than just someone to inject drugs with: Injecting within primary injection partnerships. Drug Alcohol Depend. 2015;156:275–81. Epub 2015/10/16. 10.1016/j.drugalcdep.2015.09.025 26460140 PMC4633359

[18] Gyarmathy VA, Neaigus A, Li N, Ujhelyi E, Caplinskiene I, Caplinskas S, et al Infection disclosure in the injecting dyads of Hungarian and Lithuanian injecting drug users who self-reported being infected with hepatitis C virus or human immunodeficiency virus. Scandinavian journal of infectious diseases. 2011;43(1):32–42. Epub 2010/09/16. 10.3109/00365548.2010.513064 ; PubMed Central PMCID: PMCPmc3074185. 20840002 PMC3074185

[19] Simmons J, Singer M. I love you … and heroin: care and collusion among drug-using couples. Substance abuse treatment, prevention, and policy. 2006;1:1–8. Epub 2006/05/26. 10.1186/1747-597x-1-7 16722522 PMC1524734

[20] Rhodes T, Quirk A. Drug users' sexual relationships and the social organisation of risk: the sexual relationship as a site of risk management. Soc Sci Med. 1998;46(2):157–69. Epub 1998/02/03. . 9447640 10.1016/s0277-9536(97)00156-1

[21] Rhodes T. Risk theory in epidemic times: sex, drugs and the social organisation of ‘risk behaviour’. 1997 10.1111/1467-9566.ep10934410

[22] Morris MD, Evans J, Montgomery M, Yu M, Briceno A, Page K, et al Intimate Injection Partnerships Are at Elevated Risk of High-Risk Injecting: A Multi-Level Longitudinal Study of HCV-Serodiscordant Injection Partnerships in San Francisco, CA. PloS one. 2014;9(10):e109282 Epub 2014/10/07. 10.1371/journal.pone.0109282 ; PubMed Central PMCID: PMCPmc4186818. 25286346 PMC4186818

[23] Shaw SY, Shah L, Jolly AM, Wylie JL. Determinants of injection drug user (IDU) syringe sharing: the relationship between availability of syringes and risk network member characteristics in Winnipeg, Canada. Addiction. 2007;102(10):1626–35. Epub 2007/09/15. 10.1111/j.1360-0443.2007.01940.x . 17854339

[24] Johnson RA, Gerstein DR, Pach A 3rd, Cerbone FG, Brown J. HIV risk behaviors in African-American drug injector networks: implications of injection-partner mixing and partnership characteristics. Addiction. 2002;97(8):1011–24. Epub 2002/07/30. . 12144604 10.1046/j.1360-0443.2002.00165.x

[25] Tracy D, Hahn JA, Fuller CM, Evans J, Lum P, Morris MD, et al Higher risk of incident hepatitis C virus among young women who inject drugs compared with young men in association with sexual relationships: a prospective analysis from the UFO Study cohort. BMJ Open. 2014;4(5):e004988 10.1136/bmjopen-2014-004988 24875490 PMC4039809

[26] Page K, Morris MD, Hahn JA, Maher L, Prins M. Injection drug use and hepatitis C virus infection in young adult injectors: using evidence to inform comprehensive prevention. Clin Infect Dis. 2013;57 Suppl 2:S32–8. Epub 2013/08/02. 10.1093/cid/cit300 ; PubMed Central PMCID: PMCPmc3722077. 23884063 PMC3722077

[27] Treloar C, Rance J, Bryant J, Fraser S. Harm reduction workers and the challenge of engaging couples who inject drugs in hepatitis C prevention. Drug Alcohol Depend. 2016;168:170–5. Epub 2016/10/30. 10.1016/j.drugalcdep.2016.09.010 . 27665209

[28] Syvertsen JL, Robertson AM, Palinkas LA, Rangel MG, Martinez G, Strathdee SA. 'Where sex ends and emotions begin': love and HIV risk among female sex workers and their intimate, non-commercial partners along the Mexico-US border. Cult Health Sex. 2013;15(5):540–54. Epub 2013/03/12. 10.1080/13691058.2013.773381 23473586 PMC3674135

[29] Dwyer R, Fraser S, Treloar C. Doing things together? Analysis of health education materials to inform hepatitis C prevention among couples. Addiction Research & Theory. 2011;19(4):352–61. 10.3109/16066359.2011.562619

[30] Fraser S, Treloar C, Bryant J, Rhodes T. Hepatitis C prevention education needs to be grounded in social relationships. Drugs: education, prevention and policy. 2014;21(1):88–92.

[31] Fraser S. 'It's your life!': injecting drug users, individual responsibility and hepatitis C prevention. Health (London, England: 1997). 2004;8(2):199–221. Epub 2004/04/08. 10.1177/1363459304041070 . 15068637

[32] Kelley HH, Thibaut JW. Interpersonal Relations: a theory of interdependence. New York: Wiley; 1978. 192 p.

[33] Rusbult CE, Van Lange PA. Interdependence processes. 1996.

[34] Albarracin D, Rothman AJ, Di Clemente R, del Rio C. Wanted: a theoretical roadmap to research and practice across individual, interpersonal, and structural levels of analysis. AIDS and behavior. 2010;14(Suppl 2):185–8. Epub 2010/09/09. 10.1007/s10461-010-9805-x 20824321 PMC4809135

[35] Morse JM, Stern PN, Corbin J, Bowers B, Clarke AE, Charmaz K. Developing grounded theory: The second generation (developing qualitative inquiry). 2009.

[36] Page K, Hahn JA, Evans J, Shiboski S, Lum P, Delwart E, et al Acute hepatitis C virus infection in young adult injection drug users: a prospective study of incident infection, resolution, and reinfection. The Journal of infectious diseases. 2009;200(8):1216–26. Epub 2009/09/22. 10.1086/605947 19764883 PMC2821203

[37] Strauss A., Corbin J. Grounded Theory in Practice. 1st ed Thousand Oaks, CA: Sage Publications Inc; 1997.

[38] Chamaz K. Constructing grounded theory: a practical guide through qualitative analysis. Thousand Oaks, CA: Sage; 2006.

[39] Rhodes T, Lilly R, Fernández C, Giorgino E, Kemmesis UE, Ossebaard HC, et al Risk factors associated with drug use: the importance of ‘risk environment’. Drugs: education, prevention and policy. 2003;10(4):303–29.

[40] Rance J, Rhodes T, Fraser S, Bryant J, Treloar C. Practices of partnership: Negotiated safety among couples who inject drugs. Health (London, England: 1997). 2018;22(1):3–19. Epub 2016/08/06. 10.1177/1363459316660859 . 27491943

[41] MacRae R, Aalto E. Gendered power dynamics and HIV risk in drug-using sexual relationships. AIDS care. 2000;12(4):505–15. Epub 2000/11/25. 10.1080/09540120050123909 . 11091783

[42] Riehman KS, Iguchi MY, Zeller M, Morral AR. The influence of partner drug use and relationship power on treatment engagement. Drug Alcohol Depend. 2003;70(1):1–10. Epub 2003/04/12. . 12681520 10.1016/s0376-8716(02)00332-0

[43] Morris MD, Montgomery M, Briceno A, Evans J, Andrew E, Page K, et al A study of sexual relationship power among young women who inject drugs and their sexual partners. Substance use & misuse. 2017;In Press. 10.1080/10826084.2017.1404105 PMC6063526 29286888

[44] Unger JB, Kipke MD, De Rosa CJ, Hyde J, Ritt-Olson A, Montgomery S. Needle-sharing among young IV drug users and their social network members: The influence of the injection partner's characteristics on HIV risk behavior. Addictive behaviors. 2006;31(9):1607–18. 10.1016/j.addbeh.2005.12.007 16459023

[45] Gyarmathy VA, Li N, Tobin KE, Hoffman IF, Sokolov N, Levchenko J, et al Injecting equipment sharing in Russian drug injecting dyads. AIDS and Behavior. 2010;14(1):141–51. 10.1007/s10461-008-9518-6 19214731 PMC2818991

[46] Johnson RA, Gerstein DR, Cerbone FG, Brown J. HIV risk behaviors in African-American drug injector networks: implications of injection-partner mixing and partnership characteristics. Addiction. 2002;97(8):1011–24. 12144604 10.1046/j.1360-0443.2002.00165.x

[47] Lewis MA, McBride CM, Pollak KI, Puleo E, Butterfield RM, Emmons KM. Understanding health behavior change among couples: an interdependence and communal coping approach. Soc Sci Med. 2006;62(6):1369–80. Epub 2005/09/09. 10.1016/j.socscimed.2005.08.006 . 16146666

[48] Montgomery CM, Watts C, Pool R. HIV and dyadic intervention: an interdependence and communal coping analysis. PloS one. 2012;7(7):e40661 Epub 2012/07/19. 10.1371/journal.pone.0040661 ; PubMed Central PMCID: PMCPmc3395677. 22808227 PMC3395677

[49] Hoff CC, Chakravarty D, Beougher SC, Neilands TB, Darbes LA. Relationship characteristics associated with sexual risk behavior among MSM in committed relationships. AIDS patient care and STDs. 2012;26(12):738–45. Epub 2012/12/04. 10.1089/apc.2012.0198 ; PubMed Central PMCID: PMCPmc3513980. 23199191 PMC3513980

[50] Starks TJ, Gamarel KE, Johnson MO. Relationship characteristics and HIV transmission risk in same-sex male couples in HIV serodiscordant relationships. Archives of sexual behavior. 2014;43(1):139–47. Epub 2013/11/19. 10.1007/s10508-013-0216-8 ; PubMed Central PMCID: PMCPmc3996999. 24243004 PMC3996999

[51] Darbes LA, Chakravarty D, Neilands TB, Beougher SC, Hoff CC. Sexual risk for HIV among gay male couples: a longitudinal study of the impact of relationship dynamics. Archives of sexual behavior. 2014;43(1):47–60. Epub 2013/11/16. 10.1007/s10508-013-0206-x . 24233329 PMC4425439

[52] Lewis MA, Gladstone E, Schmal S, Darbes LA. Health-related social control and relationship interdependence among gay couples. Health education research. 2006;21(4):488–500. Epub 2006/02/07. 10.1093/her/cyh075 . 16459342

[53] McMahon JM, Pouget ER, Tortu S, Volpe EM, Torres L, Rodriguez W. Couple-based HIV counseling and testing: a risk reduction intervention for US drug-involved women and their primary male partners. Prev Sci. 2015;16(2):341–51. Epub 2014/12/17. 10.1007/s11121-014-0540-9 25512179 PMC4310810

[54] Rhodes T, Rance J, Fraser S, Treloar C. The intimate relationship as a site of social protection: Partnerships between people who inject drugs. Soc Sci Med. 2017;180:125–34. Epub 2017/03/28. 10.1016/j.socscimed.2017.03.012 . 28343111

[55] El-Bassel N, Shaw SA, Dasgupta A, Strathdee SA. People who inject drugs in intimate relationships: it takes two to combat HIV. Current HIV/AIDS reports. 2014;11(1):45–51. Epub 2014/01/31. 10.1007/s11904-013-0192-6 24477931 PMC4096813

[56] Treloar C, McCredie L, Lloyd AR. Acquiring hepatitis C in prison: the social organisation of injecting risk. Harm reduction journal. 2015;12:10 Epub 2015/04/24. 10.1186/s12954-015-0045-2 ; PubMed Central PMCID: PMCPmc4413553. 25903401 PMC4413553

