# Supplementary material for: Injecting-related trust, cooperation, intimacy, and power as key factors influencing risk perception among drug injecting partnerships
Source: PLoS One. 2019 May 31;14(5):e0217811. doi: 10.1371/journal.pone.0217811 (PMC6544289; doi:10.1371/journal.pone.0217811)
Supplement: S1 Table — (DOCX) [file pone.0217811.s001.docx]

**Introductory Statement:** This is a study looking at what happens when people inject together in the same physical space. Specifically, about what happens to make people share needles, use the same spoon/baggie/cooker when mixing their drugs, or do anything else that could increase their chance of getting hepatitis C or another blood borne infection whether it is a slip up or a regular practice. **Today I’m looking to you to help me understand how people’s relationship with other people they inject with influence their injecting behaviors**. This is more like a conversation than a survey.

I am interested in both general and specific information you can offer us. To protect people’s privacy, we’re going to use nicknames throughout our conversation.

**Background questions (5min)**

***This first section is a quick survey to establish basic information.***

In the past 6 months, approximately how many different people have you injected with (in the same physical space)? _______________

In past 6 months, approximately how many of the people you counted above did you also had sex with? _______________

*Do you have someone you primarily inject with?*

Yes

No (skip to in-depth interview questions)

If yes (ask for nickname),

How long have you known [Main IP]?

In the past month, how many days have you injected drugs with [Main IP]?

____________________

When was the last time you injected with [Main IP]?

When was the first time you injected with [Main IP]?

Do you live with [main IP], that is, did you stay together in the same tent, squat, shelter, apartment, whatever for at least one night in the past month?

1 Yes

2 No

How would you define your relationship with [Main IP]? (*Mark all that apply)*

1 = Regular sex partner

**2** = Casual sex partner

**3** = Friend

**4** = Sibling or other relative

**5** = Dealer

**6** = Acquaintance

= Someone I inject drugs with

= Someone who gives me money, food, or a place to stay

= Other Specify: ____________________

***Now I am going to ask you some more open and in-depth questions.***

***The following questions will be about people you’ve injected with or about observations you’ve made about people’s injecting behaviors. Specifically their injecting behaviors when they inject with their primary (preferred) injecting partner. We’re going to refer to this type of person as the main injection partner.***

**Interviewer note: Remind the participant to use nicknames for all people discussed.

Key Question: What makes someone decide to inject with some people and not others? And what makes someone decide to inject primarily with some people but not others? (1)

What is it about someone’s **relationship** with the main person they inject with that makes them likely to share needles, cookers or rinse with him/her? (2-5)

Why do people share with some people but not others? (2-5)

Key Question: How does **trust** come into play when people inject together?

How does trust influence injecting behaviors (increase/decrease risk) when they inject with their main IP? (3)

Key Question: How does **money** influence how two people inject together?

When one person buys the drugs how does that influence how drugs are prepared? (6)

And how does it influence the other person’s ability to decide how their hit is prepared (with cleans or dirties)? (6)

When two people buy drugs together how does it affect one’s ability to negotiate injecting practices? How does that affect their ability to negotiate safe injecting practices? (6, 2)

Explain some differences in how people inject when there are only **two people** vs. **multiple** people injecting together. (2, 3, 6)

What determines how drugs are prepared when two people inject together? (2, 6)

What are some techniques you’ve seen people use to help them stay safe when they inject with other people? (1,2, 6)

What is it that makes people be able to negotiate safer injecting behaviors with some people they inject with but not others? (6)

If someone doesn’t tell you the needle you are using is a dirty is it **assumed** amongst your injecting partners that it is a clean? (2, 3)

*How can you tell it is clean?*

How does **living with someone** influence one’s injecting behaviors (sharing of needles/reusing dirties or mixing drugs together)? (1, 2, 3, 6)

What **gives** someone **power over their own individual behavior** in terms of negotiating how they want the process to go when they inject with others? (6)

What things can **reduce someone’s power** over himself or herself when they inject with others in terms of negotiating how they want the process to go? (6)

How does someone’s **power** over their drug use change when they begin to more **regularly inject** with someone (when that someone becomes their main IP)? (1,6)

Key Question: Research has shown that there is more needle sharing in injecting relationships where the people also have a **sexual** **relationship**.(1-6)

Why do you think this is?

What does your experience tell you about this?

Is there **anything else** we haven’t talked about regarding why people inject with some people and not with others? Or engage in “higher risk” behavior with some and not others? Anything regarding what affects whether or not someone share injecting equipment or does things that increase their risk for Hep C? Specifically when they inject with their main IP?
